# An Integrated Sequencing Approach for Updating the Pseudorabies Virus Transcriptome

**DOI:** 10.3390/pathogens10020242

**Published:** 2021-02-20

**Authors:** Gábor Torma, Dóra Tombácz, Zsolt Csabai, Dániel Göbhardter, Zoltán Deim, Michael Snyder, Zsolt Boldogkői

**Affiliations:** 1 Department of Medical Biology, Faculty of Medicine, University of Szeged, 6720 Szeged, Hungary; torma.gabor@med.u-szeged.hu (G.T.); tombacz.dora@med.u-szeged.hu (D.T.); csabai.zsolt@med.u-szeged.hu (Z.C.); shumbu32@gmail.com (D.G.); 2 Department of Genetics, School of Medicine, Stanford University, Stanford, CA 94304, USA; mpsnyder@stanford.edu; 3 Department of Biotechnology, Faculty of Science and Informatics, University of Szeged, 6726 Szeged, Hungary; deim.igszak@gmail.com

**Keywords:** pseudorabies virus, herpesvirus, transcriptome, Pacific Biosciences, nanopore sequencing, long-read sequencing

## Abstract

In the last couple of years, the implementation of long-read sequencing (LRS) technologies for transcriptome profiling has uncovered an extreme complexity of viral gene expression. In this study, we carried out a systematic analysis on the pseudorabies virus transcriptome by combining our current data obtained by using Pacific Biosciences Sequel and Oxford Nanopore Technologies MinION sequencing with our earlier data generated by other LRS and short-read sequencing techniques. As a result, we identified a number of novel genes, transcripts, and transcript isoforms, including splice and length variants, and also confirmed earlier annotated RNA molecules. One of the major findings of this study is the discovery of a large number of 5′-truncations of larger putative mRNAs being 3′-co-terminal with canonical mRNAs of PRV. A large fraction of these putative RNAs contain in-frame ATGs, which might initiate translation of N-terminally truncated polypeptides. Our analyses indicate that CTO-S, a replication origin-associated RNA molecule is expressed at an extremely high level. This study demonstrates that the PRV transcriptome is much more complex than previously appreciated.

## 1. Introduction

Pseudorabies virus (PRV; also called as Suid herpesvirus 1), an important veterinary pathogen, belongs to the subfamily Alpherpesvirinae of the family Herpesviridae. Its closest relatives are the bovine alphaherpesvirus type 1(BoHV-1) [1] and the varicella zoster virus (VZV) [2]. PRV causes Aujeszky’s disease [3] in swine, but other mammalian animals, such as dog, cat, sheep, cattle, and raccoon are also susceptible to the virus. Humans and horses are resistant to PRV infection. Like other herpesviruses, PRV is an enveloped virus with a nucleocapsid containing a large (~143,000 Kbp), linear, double-stranded DNA molecule [4]. Besides the lytic cycle, PRV can also enter latency mainly in the trigeminal ganglia of the infected animals [5].

PRV is a model organism for studying the molecular pathogenesis of herpesviruses, including the mechanism of neurotropism [6,7] and the regulation of gene expression. PRV also serves as a model for the development of genetically engineered vaccines [8]. Additionally, this virus is the most popular multisynaptic tracer of neural circuits [9,10,11,12]. PRV has been genetically modified in order to restrict its spread exclusively in a retrograde manner [10,13,14], to decrease its virulence [15], or to contain fluorescent protein genes for enhancing detectability of virally infected cells [16], or acting as fluorescent activity markers, which allow the simultaneous monitoring of the activity of multiple neurons using optical methods [17]. Finally, PRV has also been utilized as a vector for gene delivery to cardiac muscle cells [18] and to embryonic spinal cord graft [19], as well as an oncolytic agent [20].

The transcriptional cascade of herpesviruses was originally defined by assessing viral RNAs and proteins using inhibitors of protein synthesis (cycloheximide) or DNA replication (phosphonoacetic acid). The immediate-early (IE) genes can be expressed in the absence of de novo viral protein synthesis. The *ie180* is the only PRV IE gene, and it encodes a transcription activator [21]. Most of the E genes specify enzymes needed for the synthesis of viral DNA. The L genes code for the structural elements of the virion, including capsid and spike proteins. Late genes were further delineated as leaky late (L1) and true late (L2) based on whether they start to be expressed before or after the genome replication, respectively. Viral transcriptomes were profiled by real-time RT-PCR [22] or RNA sequencing using both Illumina-based short-read sequencing (SRS) [23,24], and long-read sequencing (LRS), including three different platforms from Pacific Biosciences (PacBio) [25,26,27], Oxford Nanopore Technologies (ONT) [27], and Loop Genomics [28]. ONT approaches included amplified cDNA, direct cDNA (dcDNA) [28], and native (direct) RNA (dRNA) sequencing [29].

Previously, we reported the characterization of the PRV transcriptome using SRS based on the Illumina platform [24] and LRS based on the PacBio RS II [26,30] and ONT MinION [31] platforms. We have developed a technique for the quantitative kinetic analysis of the PRV gene expression using LRS data [32]. In this current study, we carried out PacBio Sequel and ONT MinION sequencing using novel library preparation approaches. The number of sequence reads was significantly increased, which allowed the discovery of novel genes, transcripts, and transcript isoforms, and the confirmation of our earlier data. Additionally, PacBio Sequel and ONT dRNA sequencing generated very long reads, which helped the identification of novel ultra-long polygenic viral RNAs. In this work, we combined our novel data with the earlier data obtained using different sequencing techniques, which helped to identify novel transcripts and question the verity of some previously characterized low-abundance transcripts.

## 2. Results

### 2.1. Analysis of the PRV Transcriptome Using Sequencing Data Obtained in This and in Earlier Studies

In this work, we carried out the transcriptome profiling of pseudorabies virus using novel and formerly published sequencing data. Two LRS platforms, PacBio Sequel and ONT MinION, were applied for the generation of novel data. Oligo(dT) primers were used for the reverse transcription (RT) in PacBio sequencing, and oligo(dT) and random primers were used for the RT in ONT sequencing. Transcript identification was based on the detection of a transcription start site (TSS) and a transcription end site (TES) by at least two independent techniques using the LoRTIA software suite developed in our laboratory [33]. To identify splice sites, putative truncated mRNAs, and short 5′-untranslated regions (5′-UTR) isoforms, we applied an even more stringent criterion: Identification by at least two independent techniques plus by direct RNA (dRNA) sequencing.

Compared to our earlier publications, in this work, we used novel sequencing chemistries for dRNA and to dcDNA sequencing, the latest ONT-guppy basecaller was used instead of the earlier Albacore, and Minimap2 long-read mapper was used instead of GMAP. It is well-known that RT, PCR, and other processes can produce false TESs, TSSs, and splice sites [34] due to RNA degradation, false priming, and template switching at the repetitive sequences of the transcripts. LoRTIA software was used to eliminate spurious products and also for checking the quality of sequencing adapters and poly(A) sequences.

Direct RNA sequencing generates fewer false transcripts, but produces incomplete reads lacking 15-30 bp from the 5′ termini and 3′ poly (A) tails. Another disadvantage of dRNA-Seq is its low throughput compared to the cDNA-Seq techniques. Direct cDNA sequencing circumvents these problems, and it also eliminates non-specific events related to PCR amplification. Additionally, dcDNA-Seq produces longer reads than the amplified sequencing techniques and it yields higher coverage than the dRNA-Seq technique. Direct RNA sequencing has become the golden standard by now, because it is considered to be error-free (excluding the problems with the transcript termini). However, according to our experience, dRNA-Seq technique has its own specific biases for the generation of false transcripts, because several LoRTIA transcripts obtained using this technique were unreproducible by other techniques. Thus, the distinct techniques have different strengths and limitations, which underlines the importance for the use of multiplatform approaches in transcriptome research.

**Figure 1:**
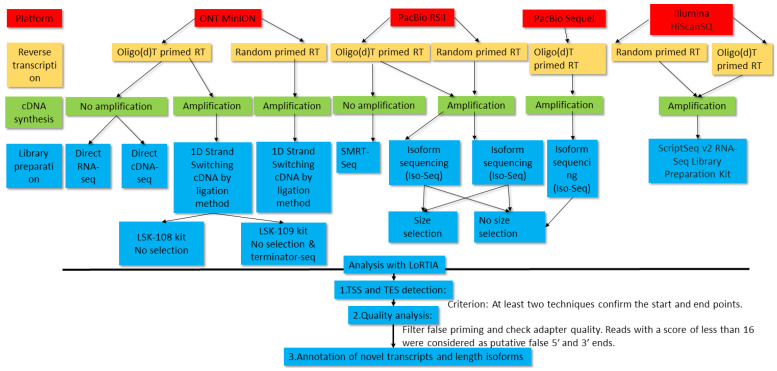
Workflow of the PacBio, MinION, and Illumina sequencing. This data flow diagram shows the detailed overview of the study design. The LoRTIA program identified the transcription start site (TSS) and transcription end site (TES) positions in Oxford Nanopore Technologies (ONT) cDNA, direct cDNA (dcDNA), Terminator-seq, PacBio RSII random, IsoSeq, and Sequel samples. LoRTIA software suite also helped in the validation of TESs and introns in dRNA-Seq samples. The Illumina data were used for the validation of low-abundance transcripts, splice sites, antisense transcription, and for the identification of transcription readthroughs.

In total, 13 ONT, 29 PacBio and 2 Illumina samples were used for the analysis of the PRV transcriptome. Figure 1 shows the workflow of experiments and bioinformatic analyses. Table S1 shows the details of transcription reads and coverages obtained by the various techniques. The detailed sequencing statistics is shown in Table 1.

**Figure 2:**
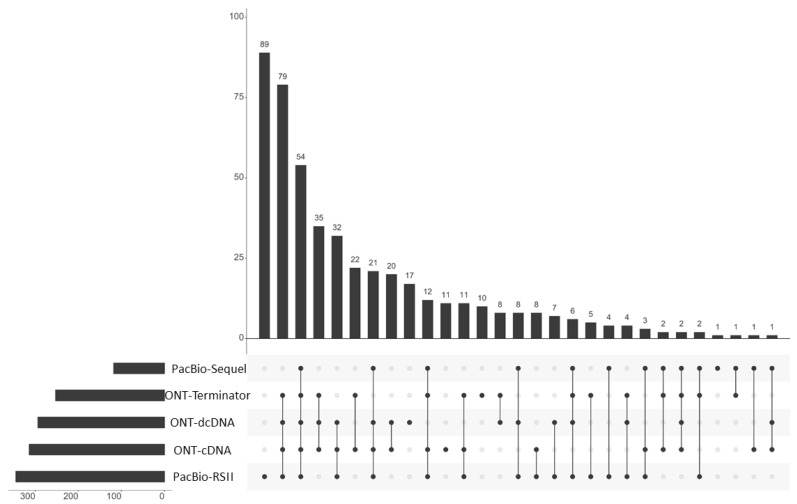
Transcript characteristics. The UpSet plot indicates the quantitative distribution of the transcripts identified by using various combinations of ONT and PacBio library preparation and sequencing techniques. The horizontal bar graph indicates the counts of annotated transcripts derived from the given sequencing approaches. The vertical bar charts show the number of transcripts (*y*-axis) detected in the various combination of sequencing approaches (*x*-axis). The black dots represent the presence of transcripts within the given experiments. Every possible intersection (lines with dots) is represented.

Using the LoRTIA suit with our stringent criteria (at least two LoRTIA transcripts obtained by different techniques), we identified 465 TSSs and 57 TESs overall. Using these TSSs and TESs, LoRTIA annotated altogether 619 transcripts of which 410 are novel (Figure 2 and Table S2)**.** Eighty-two long transcripts were annotated manually, due to their uncertain TSSs.

The cap-selection and the Terminator-based techniques in principle are able to eliminate transcripts without caps. This is because transcripts without cap will not be amplified by PCR in the first technique and these transcripts are degraded by the Terminator enzyme in the second technique.

However, the 5′-end can be lost in the next steps of processing, therefore these techniques do not provide absolute guarantee for the elimination of transcripts with false TSSs. Nevertheless, they are useful for the enrichment of full-length RNA molecules.

**Figure 3:**
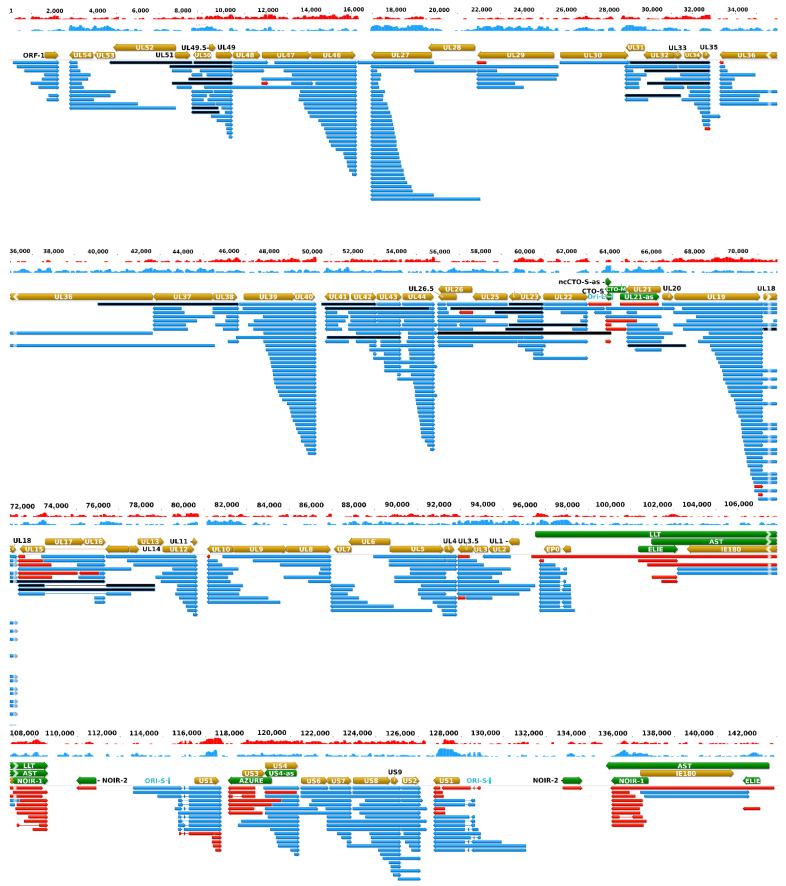
The updated pseudorabies virus (PRV) transcriptome. PRV transcriptome contains those transcripts that were identified by the integrated approach using novel and earlier short-and long-read sequencing datasets. The light brown arrows represent the open reading frames of genes; the green arrows show the non-coding RNA genes; the blue arrows are the mRNAs; and the red arrows illustrate the non-coding transcripts. Complex transcripts are also colored by black, although it cannot be excluded that they function as mRNAs. These RNA molecules contain multiple genes of which at least one is oriented in an opposite direction relative to the others. Long latency Transcript (LLT) and Antisense Transcript (AST) were detected in latently infected neurons, and by real-time RT PCR in lytic infection (especially when cells were treated with cycloheximide, a protein synthesis inhibitor [22]), but not in any of our sequencing experiments that all used lytically infected samples. The depth of viral read coverages generated by Illumina sequencing are represented by red (+ strand) and blue (−strand) colors. The coverage is plotted in a log scale using Geneious software. Table S3 contains the RPKM (Reads per kilo base per million mapped reads), and TPM (Transcript per million) values.

Strain Kaplan of PRV (PRV-Ka) is a well characterized laboratory strain. We also included strain MdBio of PRV (PRV-MdBio) [35], a recently characterized field isolate strain, into the analysis for the validation of PRV transcripts. PRV-MdBio was used for the dcDNA-Seq analysis. Figure 3 illustrates the updated PRV transcriptome, and Figure S1 indicates the relative abundance of these transcripts.

### 2.2. Novel Nested Genes

In this part of our work, we detected a total of 206 truncated mRNAs (of which 189 have not been published before) lacking a large part of their 5′-region including the ATG of the canonical open reading frame (ORF) compared to the main mRNAs with which they have co-terminal TESs. Some of these transcripts contain a shorter ORF starting with an in-frame ATG located downstream of the canonical ATG and share their stop codon with the ORF of the main transcript. These shorter nested ORFs potentially encode N-terminally truncated polypeptides. Among these 206 truncated transcripts, only 103 contain in-frame ORFs. Many of the truncated mRNAs encode the same truncated polypeptide sequence, and would be considered 5′ UTR (TSS) isoforms.

**Figure 4:**
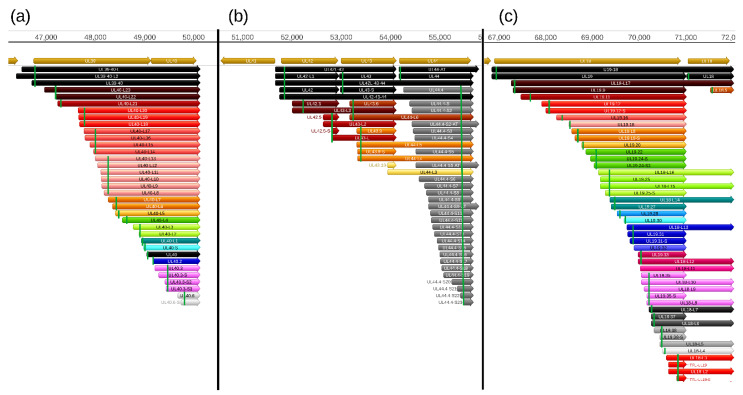
Truncated mRNAs. The following genomic regions are selected for the illustration of the truncated mRNAs: (**a**) ul39-ul40, (**b**) ul42-43-44, and (**c**) ul19-18. Arrows with the same color represent transcripts containing the same open reading frames (ORFs) but distinct TSSs or TESs. The rectangular green lines indicate the first in-frame ATGs within the transcripts. The “nc” letters at the end of the names mean ‘non-coding’, and indicate the lack of the stop codons. The light brown arrows represent the open reading frames of the PRV genes.

Additional truncated transcripts that lacking in-frame ATGs were also detected. These transcripts are presumably non-coding. Our results suggest that intra-genomic transcription initiation appears to be extremely frequent in the PRV genome. Mono- and bicistronic transcripts were detected with the same TSSs but different TESs, indicating alternate transcription termination (Figure 4, Table S4).

The stepped structure of the TSS distribution of multiple truncated RNA molecules can also be seen in Figure S2, which further suggests that they are not artifacts generated by the annotation software. The TSSs of these transcripts were confirmed by dRNA sequencing in each case. Intriguingly, the genes with a substantial intragenic TSS variety contain a large number of in-frame ATGs and practically no ATGs can be found in the other two reading frames. We searched for promoter consensus sequences, but could identify them for only a small number of truncated transcripts (Table S5). However, not all of the canonical PRV transcripts contain consensus elements, e.g., TATA-box. Additionally, TATA-less genes have been reported to be common in eukaryotic organisms [36]. The average distance between the detected TSSs and the possible upstream promoter elements, including TATA-boxes, CAAT-boxes, and GC-boxes are as follows: 34.54 bps, 118.61 bps, and. 54.46 bps for the canonical transcripts and their isoforms, and 72.6 bps, 114.75 bps, and 52.14 bps for the truncated mRNAs of PRV (Table S5). We also compared the putative downstream ATG sequences in the truncated mRNAs to the Kozak consensus sequences [37] for which the results are shown in Figure S3. It has been demonstrated that purine at −3 and G letter at +4 position display the strongest binding effect for the translation pre-initiation complex [38]. Our analysis shows that the flanking sequences of ATGs of the canonical ORFs more closely match the Kozak consensus than those of the downstream in frame ATGs of the truncated mRNAs suggesting that the canonical ORFs would be more efficiently translated.

### 2.3. Non-Coding Transcript

**Figure 5:**
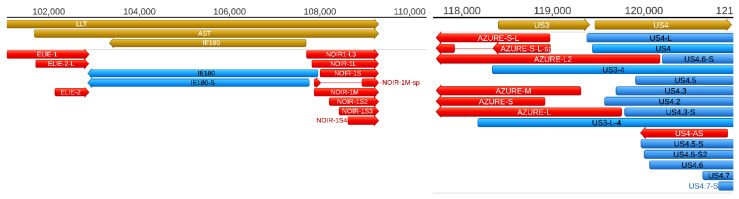
Coding and non-coding RNA molecules at the ie180-us4 genomic region. A high density of non-coding transcripts can be observed at this genomic region. Color code: Light brown: Coding, or non-coding genes, blue: mRNAs, red: ncRNAs, light brown: ORFs.

In this study, we annotated putative non-coding RNAs (ncRNAs), which all belong to the heterogeneous group of long non-coding RNAs (lncRNAs) (Table S6, Figure 5). TRL and TRS transcripts are ncRNAs at the unique long and unique short genomic region of PRV, respectively. Similar to the truncated mRNAs, these non-coding transcripts are also co-terminal with the canonical mRNAs at their TESs, but they lack a large segment at the 5′-terminal and do not contain in-frame ORFs due to the absence of ATG at this reading frame. In this work, we identified 13 novel TRLs and 7 novel TRSs. However, we could only confirm a few of the earlier published [31] putative non-coding low-abundance NCL transcripts, which are co-terminal with the mRNAs at their TSSs, but lack a certain part of their 3′-ends including the stop codons. Thus, premature transcription termination might be resulted by transcriptional noise, which produces varying 3′-termini, therefore they do not meet the stringent criteria of transcripts with well-defined termini. In this study, we only annotated two such transcripts encoded within the *ul47* and *ul26* genes.

The *ul15* gene of alphaherpesviruses has a unique structure in the sense that *ul16* and *ul17* genes are encompassed within its ORF in an opposite polarity. Earlier we have reported [26] that the downstream segment of *ul15* gene can also be separately transcribed and the resulting RNA molecule contains an out-of-frame ORF (termed fORF15). In this work, we detected novel isoforms of these transcripts.

Additionally, we also describe novel NOIR-1variants. We first identified RNAs that map to NOIR and AZURE transcripts in an earlier report [26]. Transcripts of NOIR-1 family are co-terminal with the antisense transcript (AST) and the long latency transcript (LLT) of PRV. The AZURE transcripts overlap the *us3* gene (and their long TSS variant partly the *us4* gene) in an antisense polarity. We also detected novel AZURE isoforms, including a spliced transcript. Novel splice isoforms of the NOIR-1 transcript family were also identified.

Antisense RNAs (asRNAs) were detected by both SRS and LRS in PRV. These transcripts are produced either from distinct promoters, such as the LLT, AST, and AZURE transcripts (Figure 5), or they can also be the product of transcriptional read-thorough between convergent gene pairs (tail-to-tail orientation; →←), or transcriptional overlaps between divergently arranged gene pairs (head-to-head direction; ←→). Additionally, we re-annotated the exact TSS position of US4-as antisense transcript.

The AST and LLT transcripts are included in our transcript list, however—as they are expressed in latency—we did not detect these transcripts in any of our sequencing experiments, because we used lytically infected cells in each of them. These transcripts are likely to be expressed in a very low abundance in lytic infection because we could only detect them using real-time RT-PCR [22]. Nonetheless, we could detect the NOIR-1 transcript family, which shares the TES with the latency transcripts.

### 2.4. Replication-Origin Associated Transcripts

**Figure 6:**
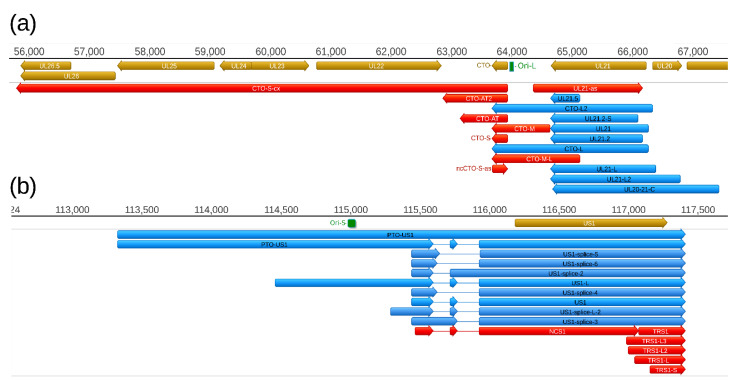
Replication origin-associated transcripts at the (**a**) Ori-L (a) and (**b**) the Ori-S genomic regions. These types of RNA molecules have been described in several viruses, including herpesviruses. Except the CTO-S transcripts, these RNA molecules overlap the replication origins through either their 3′-UTR (CTO-L), or their 5′-UTR (PTO-US1). Both the raRNAs and the transcripts of adjacent genes are overlapped by antisense RNAs of which some are controlled by separate promoters. Color code: Light brown: Coding or non-coding genes, blue: mRNAs, red: ncRNAs, light blue: Origin of replications (Ori-L and Ori-S) of the virus.

Replication origin-associated RNAs (raRNAs) have been described in several viruses, including herpesviruses [39]. First, the CTO family of PRV raRNAs has been described [30], which was followed by the discovery of the PTO transcripts [26,31] (Figure 6). In this study, we report the identification of five additional transcripts at this genomic region, including a novel TES isoform of CTO-S (CTO-S-AT2), a TSS variant of CTO-M (CTO-M/L), a very long complex RNA molecule (CTO-S-cx), and two other transcripts running with opposite polarity with respect of CTOs (CTO-as and UL21-as). While the CTO-S was found to be by far the most abundant PRV transcript, the novel RNA molecules are expressed in relative low copy numbers. Altogether, we obtained 570,653 read counts for CTO-S, whereas the second and third most abundant PRV transcripts produced only 20,453 (UL18) and 12,206 (UL16) read counts, respectively (Table S7). We also detected a splice variant of PTO-US1.

### 2.5. TSS and TES Isoforms

In this study, an even higher variety of TSSs and TESs is described compared to the earlier reports [31]. Altogether, we identified 24 shorter (not including the putative truncated mRNAs) and 166 longer novel TSS isoforms (5′-UTR variants), as well as 22 novel TES variants (3′-UTR variants) (Table S8). Due to the stringent criteria used for the annotations, the number of short TSS isoforms are likely higher than what we report here. Transcripts with longer 5′-UTR sequences than the canonical transcripts are termed by adding an ‘L’ letter to the end of the name, whereas the shorter variants are designated by adding an ‘S’ letter to the original names. However, we note that some of the long TSS isoforms that contain truncated ORFs at their 5′-UTR might be not real length isoforms. This is because if these ORFs were functional, then the downstream genes were untranslated (Figure S4). These ORFs should not be confused with the upstream ORFs (uORFs), which do not share stop codons with the canonical ORFs.

**Figure 7:**
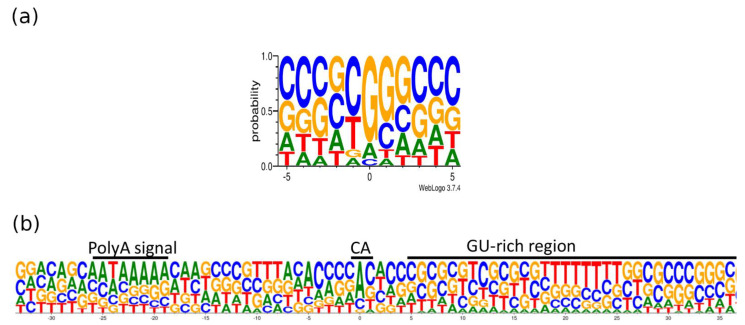
The base content at the 5′- and 3′-termini of the PRV transcripts. The *x*-axis represents the position of the nucleotides relative to the TSS (**a**) or TES (**b**), whereas the *y*-axis shows the frequency of the given nucleotide at a given position. (**a**) Most of the transcripts have GC-rich 5′ termini. The position “0” is the first nucleotide of the TSS. (**b**) Three primary sequence elements are frequent at the 3′-end of the RNAs: The hexameric polyadenylation signal (typically AAUAAA), the cleavage site (most commonly a CA dinucleotide), and the downstream sequence element (typically U/UG rich). “U”-s are shown as “T”. The position “0” is the potential polyadenylation (PA) site. The logo shows the +/− 50 bp interval of the PA site. The image is generated using weblogo 3.0 [41].

Transcripts with longer 3′-UTRs are designated by adding ‘AT’ letters to the original names. The base content at the 5′- and 3′-termini of the PRV transcripts is illustrated in Figure 7. The GGG sequence was the most common triplet at the 5′-end, whereas AU-rich sequences are found upstream and GU-rich sequences are common downstream of the poly(A) signal. It has been described that many late herpes simplex virus (HSV-1) transcripts also lack the polyadenylation signal and the canonical downstream elements, but they contain GC-rich sequences 15–20nt downstream of the cleavage site [40]. The same phenomenon has been described in bovine alphaherpesvirus (BoHV-1) [28].

### 2.6. Novel Splice Isoforms

Splicing in alphaherpesvirus transcripts is much less common than in other subfamilies of herpesviruses. Only a few PRV transcripts have been described previously [31]. In this work, we identified nine novel introns altogether (Table 2) in the transcripts EP0, US1, AZURE, NOIR-1, and PTO-US1 (Table S9). Putative introns were only accepted as true introns if their splice donor and acceptor sequences contained the canonical GT/AT or GC/AG sequences. Each splice site was validated by dRNA sequencing.

### 2.7. Novel Multigenic Transcripts

In this work, we identified 87 novel polycistronic transcripts of which 59 are bicistronic, 19 are tricistronic, 8 are tetracistronic, and 1 is pentacistronic (Table S10). The pentacistronic transcript (UL49.5-49-48-47-46) is the longest among them (7130 bps long). We also detected 24 novel complex transcripts, in which at least one of the genes stands in an opposite direction relative to the other genes. Genes oriented in a ‘wrong’ direction are presumably untranslated. The longest complex transcript is the CTO-S-cx (8135 bps), which comprises the CTO-S, *ul22*, *ul23*, *ul24*, *ul25*, and *ul26* genes of which *ul22* stands in opposite polarity relative to the others. We could not annotate the TSSs in 9 out of the 24 complex transcripts precisely, because only a few reads were obtained and because they were detected by dRNA sequencing that generates transcription reads lacking 15–20 base pairs from the 5′-ends. Although both PacBio and ONT platforms are able to read extremely long DNA stretches, it is difficult to obtain very long RNA and cDNA reads for which the reason could be the fragility of RNA molecules, the imperfectness of RT reaction, and the preference of PCR for producing for short amplicons.

### 2.8. Transcriptional Overlaps

This study revealed an even higher complexity of transcriptional overlaps than in earlier reports [31]. The transcriptional orientation of genes relative to their neighboring genes along the genome can be either convergence (→←), divergence (←→), or co-orientation (→→). Parallel and convergent transcriptional overlaps are produced by transcriptional readthroughs from tandem and convergent gene pairs, respectively, whereas divergent transcriptional overlaps are formed by the overlap of 5′-regions of RNA molecules. Tandem genes of herpesviruses are organized into gene clusters producing co-terminal transcripts as such: ‘abcd’, ‘bcd’, ‘cd’, and ‘d’ of which, according to our current knowledge, with some exceptions, only the most upstream genes are translated. Two well-described exceptions are the translation of the ORF72-71 and the ORF35-36-37 transcripts of Kaposi sarcoma-associated herpesvirus (KSHV), where the expression of the downstream genes is facilitated by an IRES sequence or an upstream (u)ORF, respectively [42,43]. The phenomenon of parallel overlaps has been long well-known. The high amount of truncated RNAs reported in this study makes the overlapping transcription patterns more complex. Our earlier studies revealed [26,31] that divergent genes also exhibit a great extent of transcriptional overlaps especially through the very long alternative 5′-UTRs. This study revealed an even higher intricacy of this type of overlap. Convergent genes are in most cases separated by relative long intergenic regions composed mainly of repetitive sequences (the only exceptions are the ul7/8, ul30/31, and ul50/51 gene pairs of which TESs are located within each other). In this analysis, using both SRS and LRS data, we demonstrated that occasional transcriptional readthroughs occur in every convergent gene pair, thereby producing antisense segments in the resulted read-through RNA molecules. An intriguing phenomenon was also observed, namely that some genes produce long TES isoform transcripts of which 3′-UTR sequences span the intergenic region but are terminated at exactly the TESs of the partner convergent genes (in UL27-AT, UL35-AT, UL44-AT, CTO-S-AT, US2-AT, Figure 3). The existence of complex transcripts might indicate an interaction between distal viral genes at the level of transcription.

## 3. Discussion

The advantage of LRS over the SRS in transcriptomics is that it can produce full-length transcripts and therefore is more valuable for the assembly of viral transcriptome than SRS, but this latter technique generates higher data coverage. Furthermore, the distinct LRS methods have different limitations and strengths. Therefore, a combination of the various approaches is advantageous for addressing the complexity of transcriptome architectures. We have developed a pipeline for the analysis of long-read RNA sequencing data. The LoRTIA software suite proved to be useful for the identification of transcript and transcript isoforms and for the exclusion of potential erroneous signals that may arise as a result of RNA degradation or during RT, PCR, and other processes.

This study delineated that the PRV transcriptome is more complex than previously anticipated. Here, we identified an unexpectedly large number of potential novel genes, which are embedded into larger host genes, and encode 5′-truncated RNA molecules many of them containing a large number of in-frame ATGs (but practically no out-of-frame ATGs in any of the other two reading frames), which may allow the translation of the truncated transcripts in a large diversity. The N-terminal truncated version of the putative proteins may have an altered effector function [44], or may play a yet uncovered role in the herpesvirus pathogenicity. The smallest truncated RNAs do not contain in-frame ATGs, therefore they are considered to be non-coding. The large number of truncated transcripts suggest a novel aspect of genomic organization, which may be not restricted to the herpesviruses, but represent a general phenomenon. In this report, we considered nested genes, which are embedded in monocistronic transcripts. However, there may be other sources for such genes. One of these sources may be the 5′-UTR sequences of transcripts, which overlap with the RNA molecules encoded by the upstream genes. In this manuscript, we consider these transcripts as TSS isoforms, but many of them may be actually bicistronic transcripts with a nested gene at the most upstream position. Similarly, the longer TSS variants of bi- or polycistronic RNA molecules may also contain nested ORFs at the most upstream positions. Hence, some of the long TSS variants of transcripts may be mistakenly designated as length (TSS or 5′-UTR) isoforms since their upstream nested ORFs may be functional. If this were the case, the genes downstream of the nested ORFs were likely untranslated. We could not identify promoter consensus sequences in many of the nested genes, but they may use the promoter of an adjacent nested gene. We found relatively weak consensus sequences for the translation initiation at the putative nested genes. However, only 40% of human genes was found to utilize highly efficient translation initiation sites (TISs) [45]. A suboptimal Kozak sequence can allow for translation machinery to skip the AUG site and start initiation at a downstream AUG codon [46]. In several PRV genes, we can find many downstream in-frame ATG triplets in close vicinity. It is possible that these genes have evolved suboptimal TISs for tuning translation efficiency low or moderate in order to be able to utilize multiple downstream AUGs start codons located on the same transcripts. Weak transcription and translation signals might result the generation of multiple transcript-length variants from a gene each with expanded coding potential. The individual putative truncated transcripts should be validated using basically different techniques, such as Northern blot analysis, whereas the coding capacity of these transcripts should be examined using e.g., ribosome profiling or Western blot analyses, or mass spectrometry. Detection of the putative overlapping polypeptides would basically redefine the herpesvirus proteome. Functional clarification of these transcripts and polypeptides would go far beyond the significance of this phenomenon in herpesviruses. However, we cannot exclude that many of these transcripts, especially the low-abundance molecules, are the result of transcriptional noise without having any function.

Intriguingly, the members of ELIE and NOIR-1 transcript families overlap the 5′- and 3′-ends of LLT/AST transcripts, respectively, but do not overlap the IE180 transcript. This form of organization suggests the ELIE and NOIR-1 transcripts do somehow interfere with transcription of the *llt* and *ast* genes, but not with the *ie180* gene during lytic infection and thereby suppressing the transcription of the two non-coding latency genes, which are normally expressed in latency. It would be intriguing to examine whether these short transcripts are active during latency, or not. We also detected novel AZURE isoforms, including a spliced variant. These transcripts have at least two TSSs that are presumably controlled by distinct promoters. The function of these putatively non-coding antisense transcripts, together with the US4-as asRNA [31], is to control the expression of *us3* and *us4* genes. The exact mechanism of this control remains to be determined [46],

Earlier we described a number of replication origin-associated RNAs in a variety of viruses [39]. The most complex pattern of these transcripts was described in PRV [26,30,31]. PRV raRNA molecules include ncRNAs, longer TSS or TES isoforms of protein-coding genes, and complex transcripts. This study revealed that CTO-S is expressed at an extremely high level compared to the other PRV transcripts. One of the proposed functions of these transcripts is to provide a replication-transcription interference mechanism that controls the initiation and the orientation of DNA replication [47]. However, since these transcripts contain poly(A) sequences, we assume that they are not a mere by-product of the above putative mechanism, but also play until now unascertained roles as RNA molecules. It would be especially intriguing to study the potential effect of the novel ncCTO-S-as on the CTO-S. Since ntCTO-S-as contains poly(A) sequences, it is likely functional as an RNA molecule, and not a mere result of the operation of a transcription interference-based mechanism.

In this and also in the previous studies, we demonstrated the existence of a vast diversity of transcription initiation from practically each herpesvirus gene. The significance of this phenomenon is currently unknown. One possibility of using multiple promoters is the differential transcription regulation of gene expression throughout the viral life cycle. Another, not necessarily exclusive explanation for the alternative TSS usage is that only the longer 5′-UTR variants contain upstream ORFs, but not the shorter ones, thereby providing additional coding potentials. The uORFs have been shown to play a role in the control of translation in multiple ways [48]. We have previously described such isoform variation first in human cytomegalovirus [49]. Additionally, long 5′-UTRs overlap with the adjacent or even more distal genes, which may lead to transcriptional interference between gene expressions as suggested before [47]. The potential function of alternative TES usage is also unknown. This phenomenon might provide an alternative regulation of transcription and/or translation.

Polycistronism is typical in prokaryotic genes but not in eukaryotes. The genetic organization of eukaryotic viruses, including herpesviruses, resemble that of bacteria in that they also express polycistronic mRNA molecules. However, polycistronism serves different functions in the two groups of organisms since at this moment we have only a few evidence for the translation of the downstream genes on a polycistronic RNA molecule in the herpesviruses [42,43]. One of the functions of this transcription system may be the (down)regulation of transcription of downstream genes by the upstream genes through interference of transcription machineries [47]. It is unknown whether complex transcripts are translated or instead they may function as lncRNAs, or maybe both.

Application of LRS techniques for transcriptome profiling revealed that the viral genomes are transcribed well beyond gene boundaries, thereby generating an intricate meshwork of transcriptional overlaps. The functionality of this phenomenon was initially regarded with skepticism. However, mounting evidence suggests that overlapping transcription fulfills regulatory purposes and provides novel strategies for the coordination of gene expression [50].

## 4. Materials and Methods

### 4.1. Cells and Viruses

PK-15 porcine kidney epithelial cell line (ATCC^®^ CCL-33™) was used for the propagation of strains Kaplan (PRV-Ka, Vanderbilt University, Nashville, TN, United States) and MdBio (PRV-MdBio, University of Szeged, Szeged, Hungary) of pseudorabies virus. Cells were cultivated in DMEM (Gibco/Thermo Fisher Scientific, Waltham, MA, United States), supplemented with 5% fetal bovine serum (Gibco/Thermo Fisher Scientific, Waltham, Massachusetts) and 80 μg of gentamycin per ml (Gibco/Thermo Fisher Scientific) at 37 °C in the presence of 5% CO_2_. For the preparation of virus stock solution, cells were infected with 0.1 multiplicity of infection [MOI = plaque−forming units (pfu)/cell]. Viral infection was allowed to progress until complete cytopathic effect was observed. It was followed by three successive cycles of freezing and thawing of infected cells in order to release of viruses from the cells. In all of the previous experiments of which data were used here, PRV-Ka was grown in PK-15 cells using the same cultivation conditions. In this work, we exclusively used lytically infected PK-15 cells for the analyses. PRV-MdBio was used for the direct cDNA sequencing, while we used PRV-Ka for the rest of the experiments. PK-15 cells were infected with 10 MOI of PRV-Ka or 1 MOI of PRV-MdBio. Infected cells were incubated for 1 h at 37 °C, followed by removal of the virus suspension and washing the cells with phosphate-buffered saline. Following the addition of new culture medium, the cells were incubated for 1, 2, 4, 6, 8, or 12 h. After the incubation, the culture medium was removed, and the infected cells were frozen at −80 °C until further use. Except the non-amplified PacBio RSII sequencing [32], where we analyzed each time point separately, in the other experiments, we used mixed time point samples (equal volumes from each sample was mixed) for the sequencing.

### 4.2. RNA Isolation

For the extraction of total RNAs, the NucleoSpin^®^ RNA II kit and NucleoSpin^®^ RNA kit (both from Macherey-Nagel) were used for SRS and LRS sequencing, respectively. In short, cells were collected by centrifugation, then the lysis was carried out by incubation in a chaotropic ion containing solution, which inactivates the RNase enzyme and provides the conditions for binding of nucleic acids to the silica membrane. ß-mercaptoethanol (3.5 µL) were also added and, subsequently, the samples were vortexed. This step was followed by the filtration of the lysate using the NucleoSpin^®^ Filter (from the kit, Macherey-Nagel, Düren, Germany) and centrifugation (1 min, 11,000 × *g*). The filters were discarded and 350 µL 70% ethanol was measured to the nucleic acid containing samples. To bind the RNA to the NucleoSpin^®^ RNA Column (part of the kit, Macherey-Nagel, Düren, Germany), the lysates were loaded onto the column and centrifuged (30 min, 11,000× *g*). Membranes were desalted by adding membrane desalting buffer (from the kit; 350 µL). This salt-removal step makes the rDNase digestion much more effective. Membranes were dried by centrifugation at 11,000× *g* for 1 min. Samples were handled with RNase-free DNase I solution (supplied in the kit) and incubated at room temperature for 15 min to eliminate DNA. Samples were washed with wash buffer (part of the RNA kit, Macherey-Nagel, Düren, Germany). Total RNAs were eluted from the membrane in a total volume of 60 μL RNase-free water. To remove the potential remaining DNA contamination, samples were handled with Ambion^®^ TURBO DNA-free™ Kit (Ambion Inc., Austin, TX, United States). In short, the RNA samples were mixed with 0.1 volume from the TURBO DNase Buffer (10×) and with the TURBO DNase enzyme (1 µL) and they were incubated at 37 °C for 30 min. The inactivation of the enzyme was carried out by adding 0.1 volume from the Inactivation buffer (supplied by the TURBO DNase kit, Ambion Inc., Austin, TX, United States). This step was done at room temperature for 5 min and then samples were centrifuged at 10,000× *g* for 1.5 min. The DNA-free RNA samples were kept at −80 °C until use. The Poly(A)+ fraction of the total RNAs were purified using the Oligotex mRNA Mini Kit (Qiagen; “Spin Columns” protocol). Briefly, the volume of the total RNA samples was set to 250 µL nuclease-free water, then 250 µl OBB buffer and 15 µl Oligotex suspension (both from the kit, Qiagen, Hilden, Germany) were measured into each of the samples. The mixtures were heated to 70 °C for 3 min and then they were cooled down and kept at 25 °C for 10 min. The mRNA-Oligotex mixtures were centrifuged at 14,000× *g* for 2 min and the supernatant were discarded. The mixtures were resuspended in 400 µL OW2 wash buffer and loaded onto spin columns (all from the Qiagen kit, Hilden, Germany) and centrifuged for 1 min at 14,000× *g*. The washing step was repeated, and finally, the polyadenylated RNAs were eluted from the membrane by adding 50 µl hot elution buffer (part of the Qiagen kit, Hilden, Germany). Half of the Poly(A)+ sample was handled with Terminator™ 5’-Phosphate-Dependent Exonuclease (Lucigen), which digests RNA with 5´-monophosphate ends but not RNAs with 5´-triphosphate, 5´-cap or 5´-hydroxyl groups starting from the 5´ end, therefore it helps to characterize 5´-ends of RNAs. The Terminator enzyme-handling of the sample was carried out as follows: Terminator 10X Reaction Buffer A, RiboGuard RNase Inhibitor and Terminator Exonuclease (1 Unit) were added to the RNA sample and then the mixture was incubated at 30 °C for 60 min. Finally, the reaction was terminated by the addition of 1 µL of 100 mM EDTA (pH 8.0). The enriched mRNA was purified using the Agencourt RNAClean XP beads (Beckman Coulter, Brea, CA, USA).

### 4.3. Pacific Biosciences Isoform Sequencing Using the Sequel System

#### 4.3.1. Synthesis of cDNAs

The cDNAs were generated from the Poly(A)+ RNA samples using the Clontech SMARTer PCR cDNA Synthesis Kit according to the PacBio Isoform Sequencing (Iso-Seq) protocol without size selection. The first-strand cDNAs were generated from the Poly(A)+ RNA with oligo(dT) primers [3′ SMART^®^ CDS Primer II A (12 µM) part of the Clontech Kit]. They were incubated at 72 °C for 3 min with slow ramp to 42 °C at 0.1 °C/s and hold at 42 °C for 2 min. 5× First-strand Buffer, DTT (100 mM), dNTP (10 mM), SMARTer II A Oligonucleotide (12 µM), RNase Inhibitor, and SMARTScribe Reverse Transcriptase (100 U) were mixed and heated to 42 °C for 1 min and then was measured into the RNA containing tube. This sample was incubated at 42 °C for 90 min, and finally the reaction was terminated at 70 °C for 10 min. These samples were amplified using KAPA HiFi Enzyme (Kapa Biosystems, Wilmington, MA, United States), according to the PacBio’s recommendations (details in our earlier publication: [50]. In sum, KAPA HiFi Fidelity Buffer (5×), KAPA dNTP Mix (10 mM), 5′ PCR Primer II A (12 µM) and KAPA HiFi Enzyme (1U/µL) were added to the first-strand cDNAs, and PCR reaction was carried out according to the following settings: Cycle the reaction with the following conditions (using a heated lid): The initial denaturation was at 95 °C for 2 min, and then 16 cycles at 98 °C for 20 s, 65 °C for 15 s and 72 °C for 4 min. The final extension was done at 72 °C for 5 min.

#### 4.3.2. SMRTbell Template Preparation for PacBio Sequel Sequencing

About 500 ng from the amplified cDNA sample was used to prepare the SMRTbell library using the Clontech SMARTer PCR cDNA Synthesis Kit (Mountain View, CA, United States) based on the PacBio Isoform Sequencing (Iso-Seq) protocol, according to our earlier publication [51]. Briefly, the cDNA damages were repaired by the addition of DNA Damage Repair Buffer, NAD+ (1mM final concentration), ATP high (1 mM final concentration), dNTP (0.01 mM final concentration), and DNA Damage Repair Mix 37 °C for 20 min (both from the PacBio Template Prep Kit, PacBio, Menlo Park, CA, United States). This was followed by the repairing of the cDNA ends by using the End Repair Mix (PacBio Template Prep Kit). The adapters were ligated to the cDNA samples with ligase enzyme (0.75 unit/µL) and ATP low was also added (0.05 mM final concentration) at 25 °C for 15 min. Finally, the exonuclease treatment was carried out (with ExoIII and ExoVII enzymes from the Template Prep Kit at 37 °C for 1 h) in order to remove the incorrect SMRTbell templates (e.g., with free ends that did not receive an adapter, or contain nicks or other damage) from the library leaving only intact SMRTbell templates. AMPure^®^ PB bead purification steps were performed after each of the enzymatic steps. The SMRTbell library was bound to the P6 DNA polymerase (Pacific Biosciences, Menlo Park, CA, United States) and annealed to v2 primers (PacBio, Menlo Park, CA, United States), then this library-polymerase complex was bound to MagBeads with MagBead Binding Kit (PacBio, Menlo Park, CA, United States). The total amount of the MagBead-bound complex was loaded onto the SMRT Cell. The MagBead One Cell Per Well protocol was used. One SMRT Cell was run on the Sequel platform.

### 4.4. Oxford Nanopore Technologies Nanopore Sequencing Using the MinION Device

#### 4.4.1. Direct RNA Sequencing

The Direct RNA sequencing (SQK-RNA002) protocol (Version: DRS_9080_v2_revM_14Aug2019) was used to obtain amplification-free transcriptomic data to remove RT and PCR biases, as well as to explore attributes of native RNA such as modified bases. Five hundred nanograms of Poly(A)+-tailed RNA was used. The library preparation was carried out according our previous publication [51] with the following modification: Agencourt RNAClean XP beads (Beckman Coulter, Brea, CA, United States) was used instead of the RNase OUT (Invitrogen)-treated Agencourt XP beads (Beckman Coulter, Brea, CA, USA).

#### 4.4.2. Direct cDNA Sequencing

Non-amplified cDNA libraries were prepared from the poly(A)+ fraction of RNAs from the MdBio strain using the ONT’s Direct cDNA Sequencing Kit (SQK-DCS109; DCS_9090_v109_revJ_14Aug2019, Oxford Nanopore Technologies, Oxford, United Kingdom) according to the manufacturer’s protocol. In brief, the Maxima H Minus Reverse Transcriptase (Thermo Fisher Scientific, Waltham, MA, United States) with SSP and VN primers (supplied in the kit) were used for the synthesis of first cDNA strand from 100 ng of poly(A)+ RNA. Next, the potential RNA contamination was eliminated using RNase Cocktail Enzyme Mix (Thermo Fisher Scientific, Waltham, MA, United States). This step was followed by the second strand synthesis using LongAmp Taq Master Mix (New England Biolabs, Ipswich, MA, United States). Double-stranded cDNA ends were repaired using NEBNext End repair/dA-tailing Module (New England Biolabs, Ipswich, MA, United States), then the sequencing adapter ligation was carried out with the NEB Blunt/TA Ligase Master Mix (New England Biolabs).

#### 4.4.3. Amplified cDNA Sequencing

ONT’s ligation-based sequencing protocol (SQK-LSK109; Version: GDE_9063_v109_revU_14Aug2019).

The ONT’s LSK109 protocol was used for sequencing the Poly(A)-selected oligo(dT)-primed, rRNA-depleted random-primed, or Terminator^TM^-handled oligo(dT)-primed samples. The usefulness of the application of Terminator^TM^ enzyme is that it enriches the capped full-length transcripts, because this enzyme processively digests the RNA molecules with 5´-monophosphate ends but not with 5´-triphosphate, 5´-cap or 5´-hydroxyl groups.

The generation of cDNA was conducted according to our previous publications [31,51] using oligo(dT) or random primers. The DNA repair was carried out according to the SQK-LSK109 protocol. Briefly, the NEBNext FFPE DNA Repair Mix and NEBNext Ultra II End repair/dA-tailing Module reagents (all from New England Biolabs, Ipswich, MA, United States) were mixed with the samples, then the mixtures were incubated at 20 °C for 5 min and at 65 °C for 5 min. This step was followed by the adapter ligation: The NEBNext Quick T4 DNA Ligase (New England Biolabs), the Ligation Buffer, and Adapter Mix (both from ONT’s Kit, ONT, Oxford, United Kingdom) were mixed with the cDNA samples and incubated for 10 min at room temperature. Samples were purified using the AMPure XP magnetic beads (Beckman Coulter, Brea, CA, United States) after each enzymatic step.

1D Strand switching cDNA by ligation method (Version: SSE_9011_v108_revS_18Oct2016) and the ONT Ligation Sequencing Kit 1D (SQK-LSK108) (ONT, Oxford, United Kingdom)

This protocol was used to analyze the random primed cDNA libraries. In short, ribodepleted RNA fraction was used to generate cDNA samples, first it was mixed with dNTPs (10 mM, Thermo Scientific) and random primers (ordered from IDT DNA) and then the mixtures were incubated at 65 °C for 5 min. After this step, the DTT and buffer form the SuperScipt IV Reverse Transcriptase kit (Life Technologies), RNase OUT enzyme (Life Technologies), and strand-switching oligo with three O-methyl-guanine RNA bases (PCR_Sw_mod_3G; Bio Basic, Canada) were added and the mixtures were heated to 42 °C for 2 min. SuperScript IV Reverse Transcriptase enzyme (200 unit) was mixed into the samples. The generation of the first cDNA strand was conducted at 50 °C for 10 min, then the strand switching step was carried out at 42 °C for 10 min. For the inactivation of the enzymes, the samples were heated to 80 °C for 10min. Samples were amplified using the KAPA HiFi DNA Polymerase (Kapa Biosystems, Wilmington, MA, USA) and Ligation Sequencing Kit Primer Mix (provided by the 1D Kit, ONT, Oxford, UK). The NEBNext End repair/dA-tailing Module (New England Biolabs, Ipswich, MA, USA) was applied to repair cDNA ends, while NEB Blunt/TA Ligase Master Mix (New England Biolabs, Ipswich, MA, USA) was used to ligate the adapters (supplied by the kit, New England Biolabs, Ipswich, MA, USA).

### 4.5. Previous Sequencing Techniques of Which Data Are Used in This Study

The methods of these approaches have been described in our earlier publications (Table 3).

#### 4.5.1. Short-Read Sequencing

Library preparation and sequencing methods were described earlier [24]. Briefly, Illumina SRS libraries were prepared using ScriptSeq v2 RNA-Seq Library Preparation Kit (Epicentre/Illumina, Madison, WI, United States) following the manual to prepare paired-end random primed libraries from ribodepleted RNAs. Single-end poly(A) sequencing was carried out from the poly(A)+ RNA fraction with custom-made anchored adaptor-primer oligonucleotides with an oligo(VN)T20 primer sequence. The Illumina HiScan SQ system was used to obtain an SRS dataset for the characterization of the viral transcriptome.

#### 4.5.2. PacBio RSII Long-Read Sequencing

The PacBio RSII instrument was used for sequencing the non-amplified [26,41] and amplified [31,41] cDNA libraries. In short, for the non-amplified method, the SuperScript Double-Stranded cDNA Synthesis Kit with SuperScript III reverse transcriptase and Anchored Oligo(dT)20 primers (both from Life Technologies, Carlsbad, CA, USA) were utilized to generate cDNAs from the poly(A)+ RNA samples. The PacBio’s DNA Template Prep Kit 1.0 following the Pacific Biosciences’ 2 kb Template Preparation and Sequencing protocol was followed as we described at the Sequel sequencing section, however P5 polymerase was used.

#### 4.5.3. ONT Long-Read Sequencing

1D Strand switching cDNA by ligation method (Version: SSE_9011_v108_revS_18Oct2016, ONT, Oxford, USA) and the ONT Ligation Sequencing Kit 1D (SQK-LSK108, ONT, Oxford, USA) were used for sequencing the Poly(A)+ transcriptome of the virus [31,41], as we described at the ‘Amplified cDNA sequencing’ section’s random-primed paragraph.

Cap-selection: The Lexogen’s TeloPrime Full-Length cDNA Amplification Kit was used to generate cDNAs (and therefore sequencing libraries) only from the capped RNAs. This method works with specific double-stranded adapters. It is required by the second strand synthesis and the adapters only ligates to the cDNAs if the inverted Gs of the cap structure are present. The cDNAs were generated from total RNA samples using the TeloPrime Kit’s buffer, reverse transcription primer, and enzyme at 46 °C for 50 min. After this step, the adapter was ligated to the cDNA in the hybrid by base-pairing of the 5′ C to the cap structure of the RNA, using a double-strand specific ligase (from the Kit, Lexogen, Vienna, Austria). The ligation was performed out at 25 °C, overnight. The second strand synthesis of the cDNA samples was carried out with the Second Strand, the PCR forward primer, and the enzyme mix (both from the Kit, Lexogen, Vienna, Austria) according to the following program: 1 cycle of 90 s at 95.8 °C, 60 s at 62 °C, 5 min at 72 °C. Finally, the double-stranded cDNAs were amplified by PCR. TeloPrime PCR Mix, PCR Forward Primer, PCR Reverse Primer, and Enzyme Mix were added. Sixteen PCR cycles of thermocycling with the following program was carried out: 1 cycle of 95.8 °C for 30 s, 50 °C for 45 sec, 72 °C for 20 min, then 15 cycles of 95.8 °C for 30 s, 62 °C for 30 s, 72 °C for 20 min, and a final extension at 72 °C for 20 min. Library generation was carried out with the ONT Ligation Sequencing Kit 1D (SQK-LSK108, ONT, Oxford, UK) as we described above, at the “1D Strand switching cDNA by ligation method” and earlier [31,41].

### 4.6. Measurement of Nucleic Acid Quality and Quantity

#### 4.6.1. RNA

The Qubit RNA BR Assay Kit (Invitrogen, Carlsbad, CA, United States) was used for the total RNA measurement, while the Qubit RNA HS Assay Kit (Invitrogen, Carlsbad, CA, USA) was applied to check the quantity of the poly(A)+ and rRNA-depleted RNA fractions. The final concentrations of the RNA samples were determined by Qubit^®^ 4.

#### 4.6.2. cDNA

The concentrations of the cDNA samples and sequencing ready libraries were measured by using the qubit dsDNA HS Assay Kit (Invitrogen, Carlsbad, CA, USA).

The RNA quality was assessed with the Agilent 2100 Bioanalyzer (for PacBio sequencing) or Agilent 4150 TapeStation System (for MinION sequencing) and RIN scores above 9.6 were used for cDNA production.

### 4.7. Pre-Processing and Data Analysis

The processing of the MinION raw data was conducted with the Guppy basecaller v. 3.6.1. with--qscore_filtering. Reads with a Q-score greater than 7 were aligned to the viral genome (NCBI nucleotide accession: KJ717942.1 [52] using the Minimap2 mapper [53]. The PacBio dataset was also mapped with Minimap2 to the same reference genome. Transcription reads were visualized using Genious 11.1.5 software (www.geneious.com, (accessed on 1 December 2020)). The upset plot was visualized by the UpSetR program [54].

Our in-house scripts (SeqTools, Department of Medical Biology, University of Szeged, Szeged, Hungary) were used to generate the descriptive quality statistics of reads (ReadStatistics, Department of Medical Biology, University of Szeged, Szeged, Hungary) and to analyze promoters (MotifFinder, Department of Medical Biology, University of Szeged, Szeged, Hungary), are available on GitHub: https://github.com/moldovannorbert/seqtools (accessed on 4 June 2020).

In this study, the LoRTIA (https://github.com/zsolt-balazs/LoRTIA (accessed on 20 August 2019)) software package (v.0.9.9, Department of Medical Biology, University of Szeged, Szeged, Hungary) was used for the detection and annotation of transcripts and transcript isoforms, as was described earlier (Table 4). Briefly, the sequencing adapters and the homopolymer A sequences were checked by the LoRTIA toolkit for the identification of TSS and TES, respectively. For the elimination of spurious TSSs and TESs (which can be caused by RNA degradation, incomplete reverse transcription and PCR, or template switching), the putative start and end sites were tested against the Poisson distribution (using Bonferroni correction). Putative introns were accepted applying the following criteria: (1) They had one of the three most frequent splice consensus sequence (GT/AG, GC/AG, AT/AC) and (2) their abundance exceeded 1‰ compared to the local coverage.

The accepted putative TSSs and TESs were considered as existing if they were identified by the LoRTIA in the datasets obtained by at least two different techniques. Potential introns were accepted as real if they were present in both dRNA-Seq and at least one of the cDNA-Seq datasets, and if they were shorter than 10 Kbps. We set a relatively low abundance for acceptance, because the proportion of splice variants may vary in different cell types, that is, rare isoforms in PK-15 cells may be frequent in other cells. The accepted TSSs, TESs, and introns were then assembled into putative transcripts using the Transcript_Annotator software of the LoRTIA suite. Very long unique or low-abundance reads that could not be detected using LoRTIA were annotated manually. These reads were also accepted as putative transcript isoforms if they were longer than any other overlapping RNA molecule. In some of these cases, the exact TSSs were not annotated. Finally, a read was considered as a transcript if it was present in at least three different samples. Transcript annotation was followed by isoform categorization according to the following principles: the most abundant transcript containing a single ORF was termed as the canonical monocistronic transcript, whereas isoforms with longer or shorter 5′-UTRs or 3′-UTRs regions than the canonical transcripts were termed TSS or TES isoforms (variants), respectively. Similarly, transcripts with alternative splicing were named splice isoforms. Transcripts with 5′-truncated in-frame ORF were termed as putative mRNAs. Transcripts containing multiple tandem non-overlapping ORFs were designated polycistronic, whereas those containing at least one ORF with opposite orientation were called complex transcripts. Transcripts with no ORFs or ORFs shorter than 30 nts were considered non-coding, except if occurred upstream of a canonical ORF in a transcript (these small ORFs were termed uORFs).

### 4.8. Accession Codes

The LoRTIA software suite and the SeqTools are available on GitHub. LoRTIA: https://github.com/zsolt-balazs/LoRTIA (accessed on 20 August 2019); SeqTools: https://github.com/moldovannorbert/seqtools (accessed on 4 June 2020).

## Figures and Tables

**Table 1:** Sequencing reads and read counts obtained by using the different techniques.

Sequencing Techniques	Number of Host Cell Reads	Viral RNA Read Counts	Average Length of Viral Reads in bp
PacBio Sequel	117,079	13,292	1553
PacBio RS II amplified	462,202	116,905	1255
PacBio RS II non-amplified	176,919	52,012	1282
PacBio-random primers	112,081	28,364	932
MinION-amplified oligo(d)T	4,273,446	1,385,284	517
MinION-non-amplified oligo(d)T	4,907,412	3,451,129	909
MinION-random	5,144,609	231,500	341

**Table 2:** Splice junction sites of the PRV transcriptome.

Transcript Name	TSS	TES	Exon 1 Position	Exon 2 Position
EP0-sp2	96,421	97,899	96,421–97,389	97,480–97,875
NOIR-1M-splice	107,880	109,304	107,880–108,010	108,932–109,304
US1-sp-2	115,437	117,407	115,437–115,591	115,713–117,407
US1-sp-3	115,437	117,407	115,437–115,638	115,931–117,407
US1-sp-4	115,437	117,407	115,437–115,621	115,922–117,407
US1-sp-5	115,437	117,407	115,437–115,632	115,922–117,407
US1-sp-6	115,437	117,407	115,437–115,765	115,922–117,407
AZURE-S-L-sp	117,716	118,967	117,716–117,917	118,340–118,967
PTO-US1-sp	113,321	117,407	115,713–115,765	1159,22–117,407

**Table 3:** List of sequencing techniques used in our previous studies.

Technique	Publication
Illumina HiScan	Oláh et al., 2015 BMC Microbiology [24]
Pacific Biosciences RSII	Tombácz et al., 2016 Plos One [26]
Pacific Biosciences RSII-dynamic	Tombácz et al., 2017 Scientific Reports [32]
Oxford Nanopore Technologies	Moldován et al., 2018 Frontiers in Microbiology [31]
All: data report, detailed protocols	Tombácz et al., 2018 Scientific Data [42]

**Table 4:** Settings of the LoRTIA software suite for each sample type.

Sample	5′ Adapter	5′ Min Score	3′ Adapter	3′ Min Score
**PacBio**	AGAGTACATGGG	16	AAAAAAAAAAAAAAA	18
**MinION cap**	default	default	default	default
**MinION non-cap**	TGCCATTAGGCCGGG	14	AAAAAAAAAAAAAAA	16
**MinION dcDNA**	GCTGATATTGCTGGG	16	AAAAAAAAAAAAAAA	16

## Data Availability

The sequencing datasets generated during this study are available at the European Nucleotide Archive’s SRA database under the accession: ERP106430 and ERP019579.

## Supplementary Materials

The following are available online at https://www.mdpi.com/2076-0817/10/2/242/s1, Figure S1: The relative abundance of PRV transcripts; Figure S2: Sequencing reads are organized in a stepped fashion indicating the separated TSSs of truncated transcripts; Figure S3: The sequence context surrounding the ATG start codon od an open reading frame; Figure S4: Putative truncated mRNAs; Table S1: Sequencing reads and coverages obtained in different techniques. Table S2: List of PRV transcripts. Table S3: The coverage of Illumina reads with the RPKM (Reads per kilo base per million mapped reads), and TPM (Transcript per million) values. Table S4: List of putative embedded genes. Table S5: List of promoter modules. Table S6: List of non-coding transcripts. Table S7: List of canonical and truncated transcripts. Table S8: List of TSS and TES isoforms. Table S9: Novel Splice isoforms. Table S10: Polygenic transcripts.
