# Supplementary figures and images for: An Integrated Sequencing Approach for Updating the Pseudorabies Virus Transcriptome
Source: Pathogens. 2021 Feb 20;10(2):242. doi: 10.3390/pathogens10020242 (PMC7924054; doi:10.3390/pathogens10020242)

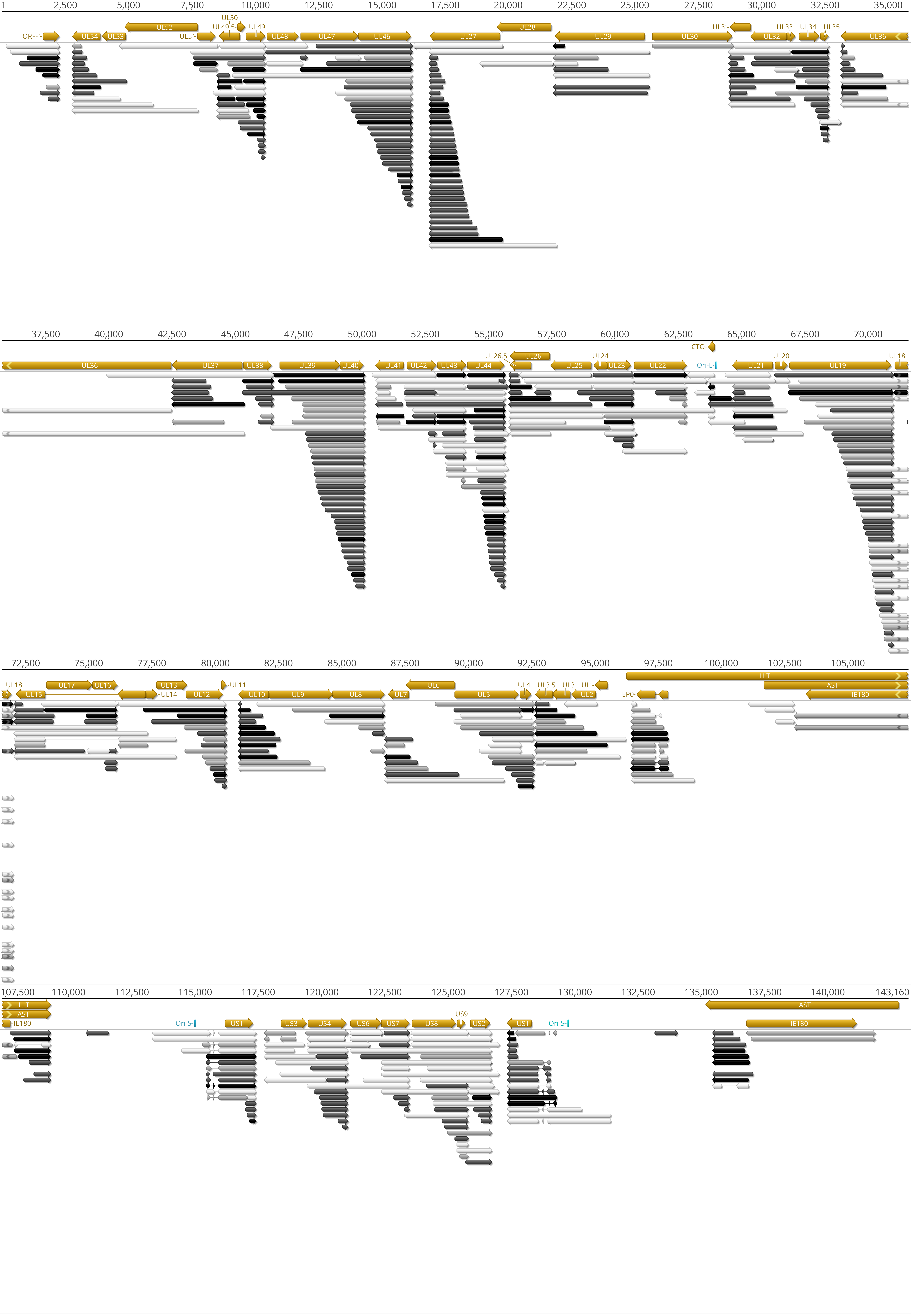

Supplement: Supplementary file 1 [file pathogens-10-00242-s001.zip › Figure S1.tiff]

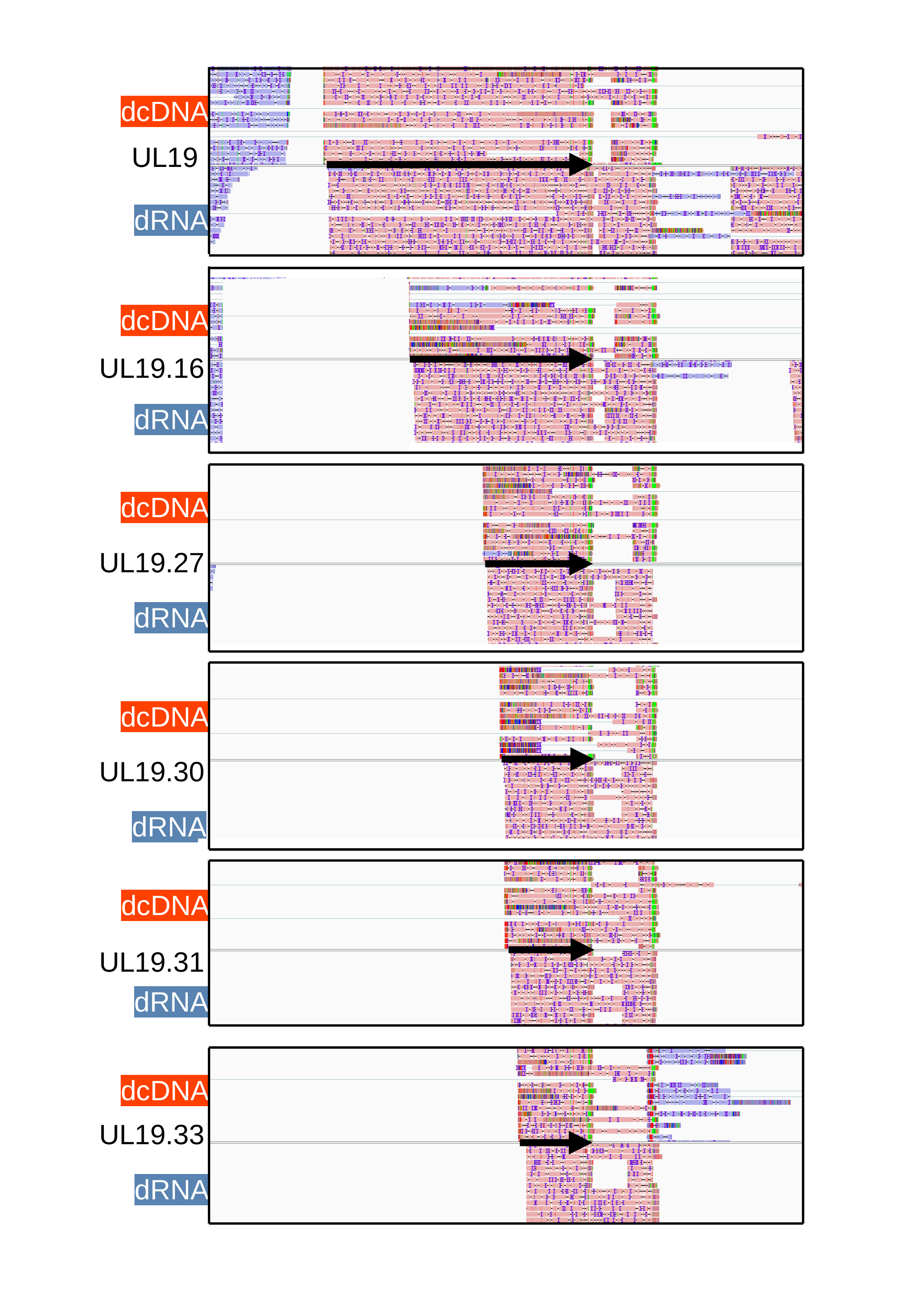

Supplement: Supplementary file 1 [file pathogens-10-00242-s001.zip › Figure S2.tiff]

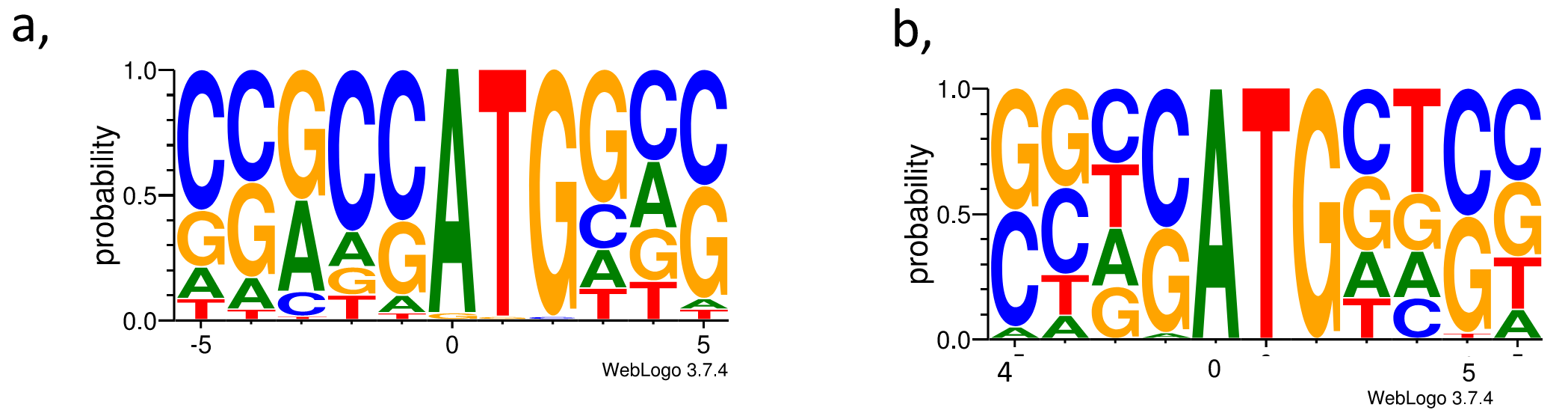

Supplement: Supplementary file 1 [file pathogens-10-00242-s001.zip › Figure S3.tiff]

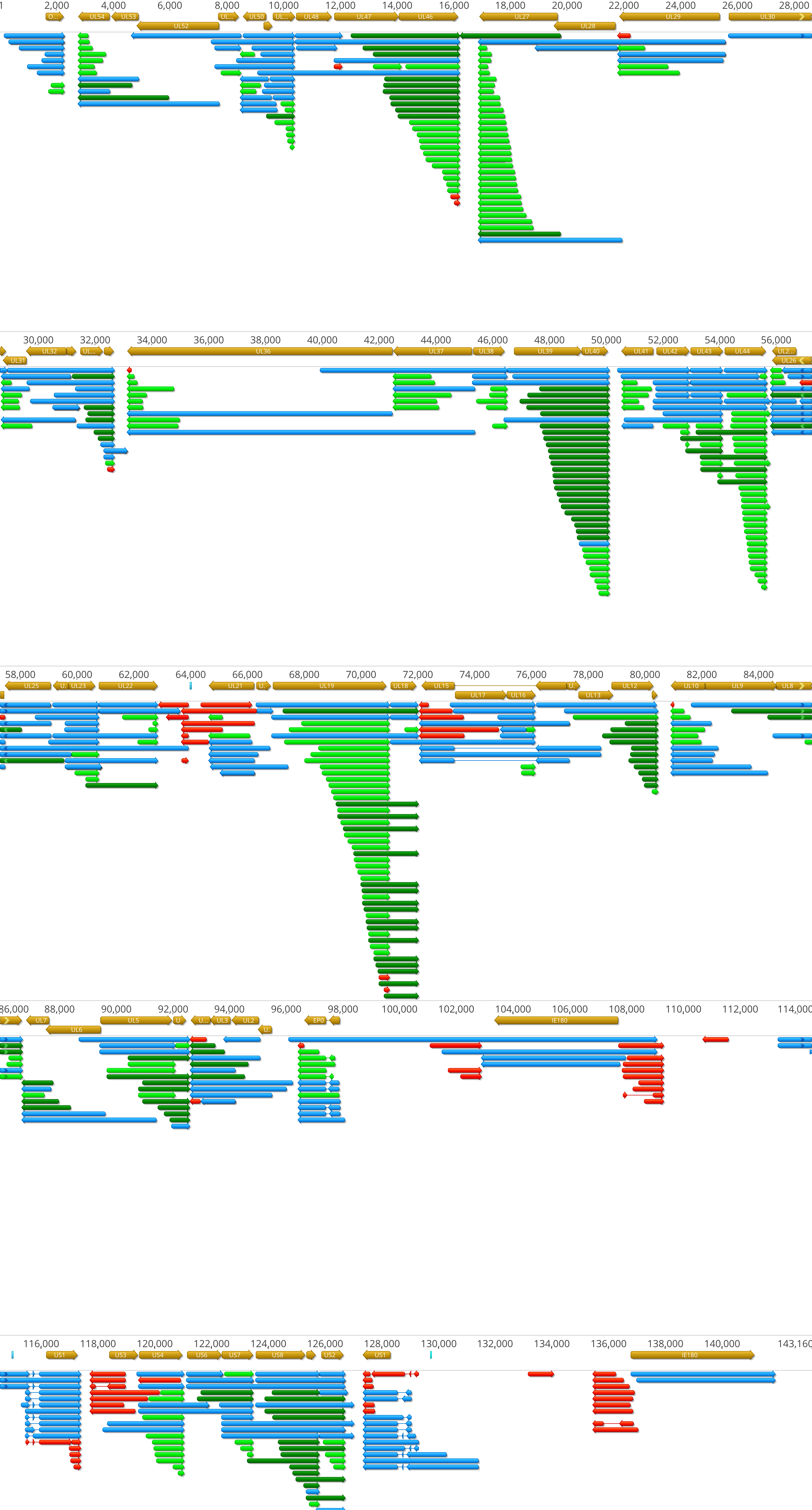

Supplement: Supplementary file 1 [file pathogens-10-00242-s001.zip › Figure S4.tiff]
